# A41 OUTCOMES FROM ESOPHAGEAL STENT PLACEMENT FOR REFRACTORY BENIGN ESOPHAGEAL STRICTURES

**DOI:** 10.1093/jcag/gwae059.041

**Published:** 2025-02-10

**Authors:** M Yau, F Donnellan, M F Byrne, S Gan, R Trasolini

**Affiliations:** Vancouver General Hospital, Vancouver, BC, Canada; Vancouver General Hospital, Vancouver, BC, Canada; Vancouver General Hospital, Vancouver, BC, Canada; Vancouver General Hospital, Vancouver, BC, Canada; Vancouver General Hospital, Vancouver, BC, Canada

## Abstract

**Background:**

A benign esophageal stricture (BES) is an esophageal narrowing that causes dysphagia in the absence of inflammation. First-line treatment for BES is esophageal bougie or balloon dilation. Esophageal stent placement is a treatment option for refractory benign esophageal strictures (RBES) but comes with significant complication risks.

**Aims:**

We aim to assess complications following esophageal stent placement for RBES and whether there are differences in migration rates depending on the stent length, stent width, and presence of clip or suture fixation.

**Methods:**

This is a retrospective cohort study of adult patients with RBES who were treated with esophageal stent placement at the Vancouver General Hospital, Vancouver, Canada between Jan. 1^st^, 2016 and Aug. 20^th^, 2024. We excluded patients with malignant esophageal strictures and esophageal leakages. Data was manually extracted via patient chart review.

**Results:**

12 patients received a total of 32 esophageal stents for RBES. 50.0% of patients had peptic strictures. 33.3% of patients had anastomotic strictures post-esophagectomy. Other causes included Barrett’s esophagus and brachytherapy. Patients received on average 7 dilations before their first stent placement. Average stent indwell time was 157 days. All stents were fully covered and metal. The average number of stents placed was 3. The average number of adverse events was 4. 87.5% of stents led to adverse events. 53.1% (17 of 32 stents) of stents migrated. 12.5% (4 of 32 stents) of stents caused granulation tissue. Stent ingrowth occurred in 6.25% of stents placed (2 of 32 stents). Other adverse events included gastrointestinal bleed, ulcers, food obstruction, dysphagia, odynophagia, chest pain, and abdominal pain. 33.3% of patients required hospitalization from adverse events. No deaths or critical care stays occurred from adverse events. 25.0% of stents were fixed with clips, padlock clips, or sutures. Risk of migration did not statistically differ between fixation and no fixation (50.0% vs 54.2%, p>0.05). Short (less than 12cm) and wide stents (greater than 18mm) were less likely to migrate as compared to long and narrow stents (52.0% vs 57.1%, p<0.05; 42.9% vs 62.5%, p<0.05; respectively).

**Conclusions:**

Esophageal stent placement is an appropriate treatment for RBES. Although adverse events occurred in most stents placed, most were mild and manageable with appropriate counselling and intervention. No adverse events occurred leading to death or critical care stay. Guidelines from the European Society of Gastrointestinal Endoscopy recommend a maximum indwell time of 12 weeks, but in select circumstances longer indwell times are possible.

**Funding Agencies:**

